# Counterproductive Academic Behaviors and Academic Performance: A Meta-Analysis and a Path Analysis Model

**DOI:** 10.3389/fpsyg.2022.893775

**Published:** 2022-06-02

**Authors:** Jesús F. Salgado, Dámaris Cuadrado, Silvia Moscoso

**Affiliations:** Department of Political Science and Sociology, University of Santiago de Compostela, Santiago de Compostela, Spain

**Keywords:** counterproductive academic behaviors, academic performance, meta-analysis, path analysis model, MASEM

## Abstract

Counterproductive academic behaviors (CAB) are a complex phenomenon that affects academic institutions in multiple geographical areas with different cultures, values, and social norms. The high incidence of CAB causes problems of critical importance that transcend the educational domain. The current study aims to contribute to the knowledge of the CAB consequences by focusing on its impact on academic performance (AP). For this purpose, a meta-analysis was conducted in order to examine the relationship between CAB, its facets, and AP. The results show that overall CAB and students' performance are negatively related with a true effect size of ρ = −0.40 (*K* = 231, *N* = 127,269). Particularly, absenteeism appeared to be the facet most strongly related to AP (ρ = −0.48, *K* = 117, *N* = 69,453). A meta-analytic path analysis model was carried out in order to test the predictive validity of CAB, students' personality characteristics, and intelligence on AP. Results show that conscientiousness and cognitive intelligence have a negative relationship with CAB (β = −0.28 and β = −0.20, respectively), and that conscientiousness, openness to experience, intelligence, and CAB can explain 58% of AP true variance. Meta-analyses of moderator variables and hierarchical meta-analyses are also presented. The implications for research and practice are discussed at the end.

## Introduction

For the past years, researchers have shown a growing interest in the study of counterproductive academic behaviors (CAB), such as cheating, plagiarism, or absenteeism. Empirical evidence suggests that CAB brings about serious consequences that affect students who engage in these behaviors, their peers, faculty, academic administration, and the educational system overall. Academic institutions are not only committed to help students obtain the best academic results through the transfer of technical knowledge and other skills, but also to reinforce students' principles, moral growth, and ethical decision making. Addressing the high incidence of CAB and taking into consideration its side effects are therefore critical issues for educational administrators, who must develop strategies to reduce CAB incident rates.

CAB is not an isolated phenomenon occurring in a specific context. It has persisted with critical incidence rates for a long period of time in academic institutions across the globe (e.g., Lim and See, 2001; Brimble and Stevenson-Clarke, 2005; McCabe, 2005; Teixeira and Rocha, 2010). Empirical evidence has also suggested that students not only engage in CAB in graduate and undergraduate degrees, but that high school and elementary school students also participate in these behaviors (e.g., Poltorak, 1995; Christensen-Hughes and McCabe, 2006; Nazir et al., 2011; Josephson Institute of Ethics, 2012; McNeal, 2014; Ison, 2015; López et al., 2016). Furthermore, this phenomenon is constantly evolving over time. New forms of CAB have appeared in recent years, potentially driven by technological developments. The research suggests that these new CAB forms occur very frequently among students and have serious consequences, much in keeping with other traditional forms of CAB (e.g., absenteeism, cheating) (McCoy, 2016; Kim et al., 2017; Aaron and Lipton, 2018).

Given the relevance of this problem and intending to make a contribution to the knowledge on CAB, the current paper presents: (1) a conceptual clarification of CAB and its facets; (2) a theoretical approach to CAB and its relationship with AP; (3) an estimation of the true effect size of the relationship between overall CAB, its facets, and AP; (4) an examination of potential moderator variables that might influence the CAB-AP relationship (i.e., type of AP indicator, AP indicator's source, and academic level); and (5) a meta-analytic path analysis model in order to determine to what extent students' likelihood to engage in CAB, their personality characteristics, and cognitive intelligence explain AP. The results are expected to be of interest for both researchers and practitioners as well as to add knowledge on the relationship between two critical constructs in educational settings.

### Counterproductive Academic Behaviors: Concept and Dimensionality

CAB can be understood as a broad construct that entails a wide variety of negative behaviors of students in academic settings. In general, research in the area has failed to provide a comprehensive definition that goes beyond the engagement in specific deviant behaviors.

CAB resembles the construct of counterproductive work behaviors (CWB) in the industrial and organizational context. Both phenomena occur in two distinct but related domains: the academic and occupational fields. Evidence on CWB sustains that this phenomenon encompasses behaviors that are volitional or intentional, that violate significant institutional norms, that harm the institution, its members, or both, and that lead to a number of undesirable consequences to the institution and others (see Robinson and Bennett, 1995; Sackett and DeVore, 2001; Gruys and Sackett, 2003). In the same vein, CAB can be understood in terms of “*any intentional behavior performed by a student that is contrary to the legitimate interests of the academic institution, its members (e.g., faculty, academic administration, other students), and the institutional goals”* (Cuadrado et al., 2021). Like CWB, these academic behaviors are counterproductive because they detract from the productive behaviors at the institution (university, high school, elementary school), disruptive because they disrupt academic-related activities, antisocial because they violate social norms, and deviant because they differ from those behaviors solicited by the academic organizations (see Ones and Viswesvaran, 2003).

Academic research has also failed to analyze the structure of CAB (Lee et al., 2020). Relying on an organizational perspective, a general construct of CAB (overall CAB) comprising different facets that group specific deviant behaviors could be proposed. Some of these are dishonest by definition (e.g., cheating on tests, plagiarism of written projects, receiving or providing unauthorized help). Others, however, do not involve deceit but are still negative, intentional, and essentially affect the educational institution's mission (e.g., unjustified absenteeism or low effort). Salgado et al. (2013) used the structure of CWB proposed by Robinson and Bennett (1995) and applied this classification to the academic context. The results showed most of the negative behaviors falling into one of the following categories: (1) cheating (cheating on examinations, plagiarism, falsification); (2) absenteeism-related behaviors; (3) stealing and damaging others' property (e.g., classmates, academic institution); (4) breach of academic rules and regulations, and (5) making a low voluntary effort on academic duties.

More recently, on the basis of this typology and after conducting an exhaustive content-analysis of the items composing the scales used to assess the negative academic behaviors in CAB research, Cuadrado et al. (2021) developed a classification comprising seven facets: (1) *cheating* (i.e., receiving or providing unpermitted help during tests or tasks that are meant to be individually completed); (2) *absenteeism* (i.e., voluntarily missing class or any other academic activity); (3) *plagiarism* (i.e., submitting another person's work as an original work or resubmitting a previous project done by the same person, as well as any other behavior that consists of the dishonest alteration of others' work); (4) *deception* (i.e., obtaining academic advantages by unethical actions such as providing false excuses or lying); (5) *breach of rules* (i.e., breaking the rules established by the instructor or the institution that define the appropriate way to behave in the academic setting); (6) *low effort* (i.e., working deliberately below one's potential); and (7) *misuse of resources* (i.e., making an improper use of the academic resources provided by the educational institution, faculty, or peers).

The consideration of CAB as a multidimensional construct is relevant for at least two reasons. First, the current fragmented status of the literature on CAB has led many researchers, such as Lee et al. (2020), to indicate that a body of research is needed where CAB is considered as an ample domain including cheating alongside other negative behaviors. Second, the analyses of the specific facets and their relationship with AP does not only add knowledge beyond the general construct of CAB but helps unmask the potential differences of the correlations at the facet level. By knowing the specific link between each CAB facet and AP, academic administrators and faculty can guide their efforts toward those counterproductive behaviors that show a greater association to AP. For instance, the measures used to reduce absenteeism might be different from those designed to control cheating on exams. In the same vein, knowing the potential differences in the results due to the moderator variables might refine strategies to fight against CAB from an applied perspective.

### Importance of Academic Performance

To examine the relationship between CAB and AP is a matter of critical importance. AP reflects any behavior under the students' control that can be graded in terms of ability, and which is relevant to their academic goals (Schmitt et al., 2008). AP, mainly expressed through grades, represents long-term work, knowledge acquisition, effort, persistence, and ability. Furthermore, it has been shown to be a valid predictor of important criteria in both the educational and the organizational domains. In the academic field, several meta-analyses showed that early AP predicts students' future academic performance and other relevant criteria such as self-efficacy, achievement motivation, financial support, retention, commitment, optimism, persistence, and self-esteem (Multon et al., 1991; Kuncel et al., 2001, 2007; Robbins et al., 2004; Grossbach and Kuncel, 2011; Richardson et al., 2012; Westrick et al., 2021). In the occupational context, AP has been shown to correlate with job performance, work satisfaction, training proficiency, and salary among other measures of occupational success (Cohen, 1984; Hunter and Hunter, 1984; Dye and Reck, 1989; Roth et al., 1996; Roth and Clarke, 1998; Strenze, 2007). This construct has also shown to be a reliable indicator of academic success both for applied and scientific purposes. This is true whether grades are obtained from academic registers or if they are self-reported by students (see Kuncel et al., 2005a; Bacon and Bean, 2006; Beatty et al., 2015). Indeed, AP outcomes (e.g., GPA) are used as a criterion for admission to universities, specific academic courses, master' degrees, PhD programs, and occupational internships. Recruitment professionals also consider AP indicators for hiring decisions (Rynes et al., 1997; Roth and Bobko, 2000).

In summary, empirical evidence places AP as a valid and reliable indicator of academic success that is appropriate for decision making in educational and occupational settings. Thereby, given the importance of AP, both theoretically and practically, it is a major concern for researchers and practitioners to determine the extent to which it can be linked to the occurrence of CAB.

### The Relationship Between CAB and AP: Empirical Evidence

Empirical evidence published so far has shown a negative association between CAB, its facets, and AP. The narrative reviews by Bushway and Nash (1977), Crown and Spiller (1998), Cizek (1999), and Miller et al. (2007) concurred that CAB is negatively linked to AP. A meta-analysis by Whitley (1998) also revealed a negative correlation between CAB and AP. The observed effect sizes ranged from *r* = −0.16 for the relationship between CAB and students' GPA to *r* = −0.36 when the criterion variable was the score obtained in laboratory tasks. More recently, Lee et al. (2020) also found a negative link between a subset of CAB behaviors, mainly cheating and plagiarism, and GPA (ρ = −0.17).

When the specific facets of CAB are independently considered, the results emerge in the same direction. For example, Credé et al. (2010) found that, regardless of the academic performance measure examined, absenteeism and AP were negatively related. The effect sizes were ρ = −0.44 with GPA and ρ = −0.41 with course grades. More recently, Gubbels et al. (2019) reported a mean effect size of *r* = −0.23 between academic achievement and school absenteeism. Primary research also reports consistently a negative relationship between AP and low effort behaviors, cheating, deception, breach of rules, plagiarism, and misuse of resources (Antion and Michael, 1983; Blickle, 1996; Tibbetts, 1999; Tate et al., 2007; Sheets and Waddill, 2008; Anderman et al., 2010; Tadesse and Getachew, 2010; Ziegler and Stoeger, 2010; DeRosier and Lloyd, 2011; Peklaj et al., 2012; Hensley et al., 2013; McDonald, 2013; Skaar and Hammer, 2013; Rickert et al., 2014; Cuadrado et al., 2019).

### Limitations of Previous Research on the CAB-AP Relationship

The meta-analyses carried out by Whitley (1998), Credé et al. (2010), Gubbels et al. (2019), and Lee et al. (2020) made unique contributions to the study of academic counterproductivity and helped to create a better understanding of the empirical association between CAB and AP. However, they present some aspects that are expected to be improved in the current meta-analysis. The study by Whitley (1998) is, to date, the only meta-analysis examining the relationship between overall CAB and AP. However, only a limited part of CAB (i.e., cheating and plagiarism) was analyzed. As a consequence, the compound does not fully represent CAB's variance. Second, different indicators of AP were separately assessed. Although very interesting results appeared, the extent to which overall AP relates to CAB needs to be tested. Third, the data search ended in 1996. Since then, a good number of studies has been conducted and need to be accumulated in order to provide an updated estimate of the relationship. Fourth, the study consists of a bare-bones meta-analysis, meaning that no artifactual corrections were applied. This is a critical issue given the fact that artifactual errors distort findings. The need to correct errors such as lack of reliability and range restriction has been widely recognized in scientific literature (see Hunter et al., 2006; Hedges, 2009; Matt and Cook, 2009; Schmidt and Hunter, 2015; Le et al., 2016). Furthermore, because meta-analytic estimates are used as input data in path modeling, the lack of corrections also negatively affects the development of a valid cumulative knowledge (Schmidt et al., 2009). Fifth, the samples accumulated belong exclusively to the North American college context. A meta-analysis of studies from other countries and academic levels is also needed. Last, the number of studies and the total sample size of Whitley's meta-analysis are relatively small.

In addition to the general CAB-AP relationship, the estimation of the effect size between AP and the specific CAB facets is essential because the results might be masking potential differences at the facet level. This will also allow researchers and practitioners to improve the precision and efficiency of the decision making for scientific and applied purposes. To this concern, prior meta-analyses have only examined one CAB facet: absenteeism. Credé et al. (2010) studied the absenteeism-overall CAB relationship and Gubbels et al. (2019) focused on the absenteeism-GPA/course grades link. However, these integrations present three limitations. First, the meta-analysis by Credé et al. (2010) was published nearly one decade ago, meaning that most recent research on the topic needs to be accumulated. Second, both meta-analyses focus on restricted contexts and samples—Credé et al. (2010) on college samples and Gubbels et al. (2019) on a compound of high school and elementary school samples from Western countries. Third, artifactual corrections are neglected in the meta-analysis by Gubbels et al. (2019) and only partially applied (i.e., measurement error in Y) in the meta-analysis by Credé et al. (2010).

Last, the meta-analysis carried out by Lee et al. (2020) provides an interesting and up-to-date review on the CAB-AP relationship. However, only a limited part of CAB (mainly cheating and plagiarism) is considered, AP is essentially assessed through students' GPA, the various CAB facets are not separately analyzed, and it only considers samples composed of college students mainly from the North American context. Last, range restriction corrections are not applied.

### A Theoretical Approach to Counterproductive Academic Behavior and Its Relationship With Academic Performance

To date, research on CAB has mainly focused on the study of issues such as the rates of prevalence, the potential antecedents of CAB, or the effectiveness of deterrence strategies against this phenomenon (e.g., Whitley, 1998; McCabe, 2005; Malgwi and Rakovski, 2009; Teixeira and Rocha, 2010; Paulhus and Dubois, 2015; Sattler et al., 2017). Research has also examined the CAB-AP relationship. However, the main characteristic of these studies is that they have been atheoretical, meaning they were non-theory-based. To overcome this limitation, we present a theoretical approach to the CAB-AP relationship based on the empirical findings of previous primary and meta-analytic studies, on conceptual rationales, as well as on theoretical approaches used in other research domains.

As suggested, CAB could be included in the same category as other negative behaviors like CWB, medical assistance fraud, medical recipe fraud, social security fraud, tax evasion, white-collar offenses, and other more general deviant business practices. The main objective of this set of behaviors is to achieve some advantage, which would otherwise be impossible or less probable to achieve (or which would require the investment of more resources) by using deviant, unethical, or unfair procedures. Consequently, some of the theoretical approaches suggested to explain those behaviors can be useful in explaining CAB and its relationship with academic performance and other academic criteria. Next, the key points of this theoretical approach are presented.

First, this theoretical approach sustains that CAB is a phenomenon hierarchically organized in a three-stratum structure. In the lower level, there is a wide range of distinct but closely related behaviors. In the second stratum there are seven facets or dimensions (i.e., absenteeism, low effort, cheating, deception, plagiarism, breach of rules, and misuse of resources). Each facet includes several behaviors. For instance, absenteeism includes lack of attendance, tardiness, and early leave without a fair excuse. The facets are moderately-to-highly related among themselves. Finally, at the apex of the structure, there is a general factor of CAB that explains the relationship among the facets. Several empirical studies support this structure (Choragwicka, 2014; Salgado, 2014; Cuadrado et al., 2021). This three-stratum structure is similar to other three-stratum structures found, for instance, in the cognitive ability domain (e.g., Carroll, 1993), in the personality domain, such as the Five-Factor model (e.g., Costa and McCrae, 1992) and the HEXACO model (Ashton and Lee, 2007), and in the job performance domain (e.g., Viswesvaran et al., 2005). The relationships between the CAB facets and the results of exploratory and confirmatory factor analyses supporting this three-stratum hierarchy are presented in Supplementary Tables 1–5.

Second, partially based on the assumptions of the prospect theory (Kahneman and Tversky, 1979, 1984; Tversky and Kahneman, 1992), the current theoretical approach sustains that CAB is based on student's voluntary decisions that imply risk and uncertainty. The application of the prospect theory to CAB implies that students would engage in these behaviors depending on whether the expected outcome is positive (e.g., to pass a course, to get better grades, to get a higher GPA) or negative (e.g., to fail a course or an examination, to get lower grades). If students perceive that the totality of the work and duties made up until that moment is higher than the amount of work and activities that they must do to pass the examination or the course, they will expect to obtain a gain by acting honestly. However, if students perceive that the work performed is lower than that which is required to pass, they will perceive it as a loss of time and effort. Therefore, according to the prospect theory, if students expect to pass, they will feel risk aversion and would avoid committing CAB. On the other hand, if students expect to fail, they would be more prone to risk (and to commit CAB) to minimize the probability of failure. Additionally, two relevant assumptions of the prospect theory are the probability of getting caught (audit principle) and the punishment (penalty) rate. According to the first assumption, if students commit CAB, there is a probability that they will get caught. According to the second assumption, if students are caught, they will be penalized. The punishment is mainly determined in the norms set by the academic institution and, partially, by the instructors (i.e., the individual tolerance). The probability of getting caught depends fundamentally on the intensity of the CAB control (i.e., CAB audit) and, to a lesser extent, on the amount of CAB. The intensity of control, defined as the individual perception of the difficulty to carry out a particular behavior, has also been proposed by the theory of planned behavior (Ajzen, 1991) as a direct determinant of the conduct. If individuals believe that they will be successful in CAB without being discovered and that CAB will be followed by a good outcome (e.g., better grades), the probability to engage in CAB will be higher.

Third, the current approach sustains that CAB is a complex phenomenon with a diversity of causal antecedents. The two main personal determinants are personality (i.e., conscientiousness and agreeableness) and cognitive intelligence. These two individual differences variables, together with psychosocial determinants (e.g., group norms, social stigma) and situational characteristics (e.g., course, instructor, task difficulty, in-class vs. online course), are the determinants of individual CAB. Supporting this approach, Cuadrado et al. (2021) found that personality and cognitive intelligence correlate with CAB at all educational levels (i.e., university, high school, and elementary school). On the other hand, some findings based on the general theory of crime (Gottfredson and Hirschi, 1990) indicate that students engaging in CAB would be individuals with low impulse control. Gottfredson and Hirschi (1990) suggest that some cognitive abilities and some facets of the Big Five personality model determine disruptive behaviors: (1) impulsivity; (2) preference for simple tasks that do not require complex cognitive processes; (3) risk-seeking; (4) preference of physical over cognitive; (5) egoism or self-centeredness, and (6) facility to lose one's temper.

Fourth, this theoretical approach posits that the facets and the general CAB factor are related to various academic criteria, including student's performance, faculty's motivation, team performance, academic climate and culture, and academic sustainability. It also argues that the general CAB factor is related to all the academic criteria. However, the CAB facets would be only related to some criteria. The approach also sustains that, when the facets are related to the same criterion, they are not related by the same extent. In other words, the approach expects some differences in the magnitude of the relationships between the facets and the criteria. In addition, the main force explaining the relationship between the facets and the criteria is the general CAB factor-facet relationship. In other words, the validity of the facets is mainly due to the variance of the general CAB factor contained in the facet.

Fifth, in line with the empirical results found in the CAB literature (e.g., McCabe et al., 1996; McCabe and Treviño, 1997; Hensley et al., 2013; Holtrop et al., 2014), the theoretical approach sustains that CAB produces lower academic results and negative outcomes due to numerous and varied potential reasons that are presented in Table 1.

**Table 1:** Rationale supporting a negative relationship between CAB and AP.

**List of propositions**	
1	CAB reduces the possibility to learn and to acquire new knowledge on the concepts taught by the instructors
2	It reduces the amount of time spent studying
3	It reduces the amount of personal work undertaken in a course
4	It hinders students from using this knowledge in future courses, discussions, and exercises
5	It increases the amount of effort needed to understand instructor's explanations
6	It reduces the capacity to understand key concepts
7	It limits class participation, which subsequently does not allow students to ask questions to clarify key concepts and obscure points
8	It limits the participation in small group activities such as class discussions
9	It obligates instructors to repeat some material or lessons, wasting class time and resources that could be devoted to other topics
10	It hinders students from taking their own notes to facilitate knowledge acquisition
11	It impedes the evaluation of the students' class participation, leading to a negative evaluation when this aspect is graded
12	It makes the interaction with teachers and other students more difficult or impossible
13	It limits the coordination with faculty and other students
14	It limits the opportunity to listen to the instructor's responses to peers' questions
15	It limits the possibility to organize one's own lines of thinking in comparison with others' new ideas and ways of thinking
16	It reduces the quality of the work and assignments done
17	It reduces student's attention during class time
18	It provokes class time interruptions
19	It reduces students' motivation by affecting their goal setting
20	It makes the acquisition of tacit knowledge, values, and contextual learning more difficult or impossible
21	It may lead to an early abandonment of educational institutions
22	It impedes or limits assignments to be turned in on time;
23	It reduces students' self-confidence and self-esteem regarding their own knowledge
24	It produces feelings such as stress, frustration, and low self-efficacy in teachers
25	It demotivates teachers and disincentives their efforts
26	It limits the possibility of others to use the material and resources (by robbery or property damage)
27	It generates emotions like anger, skepticism, and pessimism when honest students see their dishonest counterparts achieving academic benefits through the use of fraudulent means
28	It can produce additional costs that are compensated by student fees (academic institutions will have to buy new copies of textbooks or resources, which also requires additional clerical work)
29	It negatively affects the academic institutions' climate and culture by enhancing an atmosphere of permissiveness and acceptance when negative behaviors are not addressed
30	It devaluates the educational system
31	It tarnishes academic institutions' reputation
32	It produces negative effects on academic sustainability
33	It impacts organizational sustainability, as students engaging in counterproductive academic behaviors are more prone to engage in counterproductive work behaviors

Sixth, this approach also suggests the application of some mechanisms to reduce rates of CAB and its effects and, consequently, to improve academic performance. The three main strategies are: (a) to increase the control of this phenomenon with an expansion of the number of procedures that impede and limit CAB; (b) to increase the fear of getting caught through an escalation of severity in punishment, and (c) to reduce instructors' tolerance of CAB. Some studies found that fear of punishment is the most effective way to limit CAB (e.g., Genereux and McLeod, 1995; Diekhoff et al., 1996, 1999; Burns et al., 1998; Vandehey et al., 2007). However, the fear of punishment is affected by the perception of the likelihood of getting caught in CAB engagement (e.g., cheating, plagiarism). These two mechanisms (an increase of punishment and of CAB control) must operate together. Instructors' tolerance of CAB produces lower academic results by reducing students' fear of penalty, thus facilitating the probability to commit CAB. This tolerance can also generate passivity in other instructors and students in the institution. In the discussion, we will offer some specific actions aiming to control CAB.

### Meta-Analytic Path Analysis Model of CAB-AP Relationship

Based on some of the propositions included in the theoretical approach presented above and on the findings of previous research, we have developed a meta-analytic path analysis model of the CAB-AP relationship. Particularly, we focused on a group of variables categorized as individual differences: the students' personality characteristics, following the Big Five model of personality (emotional stability, extraversion, openness to experience, agreeableness, and conscientiousness), and cognitive intelligence. The selection of this set of variables is based on the empirical evidence that has been published in the previous decades. Research has consistently pointed to the existence of certain individual characteristics as the best predictors of AP. Among them, some of the Big Five dimensions and cognitive intelligence have been extensively shown to be excellent predictors of individual performance in academic settings. Consequently, testing their joint effects to explain both the tendency to engage in CAB and AP will generate original results and further the knowledge on individual performance in academic contexts.

In regard to the personality dimensions and their relationship with AP, conscientiousness stands out among the others in predicting academic success. Individuals scoring higher in this dimension are organized, work-oriented, persistent, and self-disciplined (Goldberg, 1990, 1992; Costa and McCrae, 1992; Saucier and Goldberg, 2002; DeYoung et al., 2007). In the academic domain, these traits typically define high academic achievers as they represent the characteristics needed to overcome academic challenges. Accordingly, De Raad and Schouwenburg (1996) state that conscientiousness is conceptually the closest dimension of the Big Five model to school attainment. In the last decades, multiple meta-analyses found that conscientiousness is a predictor of AP (Trapmann et al., 2007; Poropat, 2009, 2014; Richardson et al., 2012; McAbee and Oswald, 2013; Salgado and Táuriz, 2014). Given the fact that conscientious students tend to be those who achieve better academic outcomes, it could be also expected that they do not have the need to act in a deviant manner to get higher grades. This argument could support the negative relationship between conscientiousness and CAB. A second explanation backing the conscientiousness-CAB negative link is that conscientious individuals are responsible, have a profound sense of duty, and are compliant with rules. These are traits that might make them reluctant to engage in negative behaviors. Consistent with the previous arguments, meta-analytic evidence places conscientiousness as the best predictor of counterproductivity in the academic context when compared to the remaining dimensions of the Big Five model (Credé et al., 2010; Giluk and Postlethwaite, 2015; Cuadrado et al., 2021). These three arguments agree with the theoretical approach presented above.

A second factor of personality that has been successfully linked to CAB is agreeableness. This dimension is comprised of characteristics like tolerance, cooperation, sympathy, or tenderness (Goldberg, 1990, 1992; Costa and McCrae, 1992; Saucier and Goldberg, 2002; DeYoung et al., 2007). These traits could describe an individual who easily adjusts to social contexts like academic settings. Agreeable people are also honest, straightforward, have a sense of morality, and reject obtaining advantages at the expense of others. Accordingly, one could easily infer that agreeable students are more averse to participating in CAB than students with lower scores in this dimension. Indeed, meta-analytical evidence on this relationship has supported the negative association between the variables (Giluk and Postlethwaite, 2015; Cuadrado et al., 2021). Again, this rationale agrees with the theory.

In the prediction of AP, openness to experience is another dimension of the Big Five model positively linked to academic achievement. Individuals who are opened to experience are creative, imaginative, aesthetically sensible, and pay attention to internal feelings (Goldberg, 1990, 1992; Costa and McCrae, 1992; Saucier and Goldberg, 2002; DeYoung et al., 2007). Furthermore, they are intellectually curious and learning oriented. These traits predispose individuals to seek knowledge and make the most of opportunities provided in the academic context. These traits could also be the link between openness to experience and AP. Certainly, meta-analyses on the topic have positively linked both constructs (Trapmann et al., 2007; Poropat, 2009, 2014).

In summary, some of the Big Five dimensions have shown to be correlated to CAB and AP. Conscientiousness and agreeableness appear to predict CAB while openness to experience and, especially, conscientiousness are linked to AP.

Together with these personality factors, cognitive intelligence emerges as the strongest predictor of academic achievement. As Jensen (1998) pointed out, if there is any unquestionable fact in applied psychometry, it is that cognitive intelligence tests have an unequivocal high degree of predictive validity for many educational criteria such as school and college grades, graduating on time, the probability of entering college and, after entering, the probability of receiving a bachelor's degree. Broadly, cognitive intelligence refers to the fundamental ability to reason (Cattell, 1943; Carroll, 1993; Jensen, 1998). Those high in cognitive intelligence do not only assimilate training better, but also learn more effectively from experience. Cognitive intelligence is also linked to knowledge and information processing, two types of ability closely related to the academic activity (Kuncel et al., 2004). Accordingly, meta-analyses have shown that cognitive intelligence is a construct with a strong and positive association to the accomplishment of academic goals (see Kuncel et al., 2001, 2010; Strenze, 2007; Postlethwaite, 2011; Salgado and Moscoso, 2019). Regarding CAB, it could be expected that students with higher cognitive intelligence are not tempted to engage in prohibited activities to accomplish school goals, because they are those most likely to succeed in class. Hence, a negative relationship between the constructs could be expected. A second argument sustaining this negative association is that proposed by Dilchert et al. (2007). As these authors suggest, one possible mechanism that might explain the link between cognitive intelligence and deviancy is the difficulty for those scoring lower in intelligence to envision the consequences of their behaviors. From this perspective, students scoring higher in cognitive intelligence would be aware of the negative consequences of being discovered engaging in CAB (i.e., getting a reduction on a test score, an academic expulsion, or other disciplinary sanctions). This anticipatory ability would prevent them from engaging in CAB. Different meta-analyses on the topic supported the negative association between the variables (Credé et al., 2010; Paulhus and Dubois, 2015; Cuadrado et al., 2021).

In short, a bulk of empirical evidence suggests that some of the Big Five dimensions of personality and cognitive intelligence account for CAB and AP variance. Following the theoretical rationale and the empirical evidence described above, the model tests CAB and AP, two criteria that are preceded by the following variables: (1) conscientiousness, agreeableness, and cognitive intelligence in the case of CAB; and (2) openness to experience, conscientiousness, and cognitive intelligence in the case of AP. The model also proposes conscientiousness and cognitive intelligence as indirect predictors of AP through overall CAB.

### Research Goals and Hypotheses

The current research aims to broaden the knowledge on the association between CAB and AP. Thus, after presenting a novel theory on this relationship, our main goals are: (1) to provide researchers and practitioners with an accurate estimate of the true relationship between CAB and AP by using methods of psychometric meta-analysis with artifactual corrections; (2) to meta-analytically estimate the extent to which the CAB facets correlate to AP; (3) to examine whether the type of measure used to assess AP, the source of the AP indicator, and the students' educational level moderate of the CAB-AP relationship; (4) to carry out hierarchical meta-analyses that give information on potential and previously undetected relationships and (5) to test a meta-analytic path analysis model of the validity of students' engagement in CAB, personality, and cognitive intelligence to predict AP. More specifically, on the basis of the theory presented above and, on the findings previously discussed, the current study is guided by the following hypothesis and research questions:

Hypothesis 1: Overall CAB and its facets correlate negatively with AP.

Research question 1: How do the CAB-AP relationship varies across the CAB facets?

Research question 2: Does the type of AP measure moderate the CAB-AP relationship?

Research question 3: Does the source of the AP indicator moderate the CAB-AP relationship?

Research question 4: Does the students' academic level moderate the CAB-AP relationship?

## Methods

### Literature Search

We conducted a comprehensive search of studies that linked any counterproductive academic behavior committed by students from any country at three educational levels (elementary school, high school, and higher education) with any measure of academic performance. For this, we carried out an electronic search in ERIC, JSTOR, PsycINFO, ScienceDirect, SpringerLink, Taylor and Francis, Teseo, and Wiley Online Library databases, and used both Google and Google Scholar engines. The search was done using every possible combination of the terms *counterproductive academic behavior, academic dishonesty, academic misconduct*, and *academic integrity* with *academic performance, grade point average* (*GPA*), and *grades* [e.g., (“academic dishonesty” and “academic performance”), (“academic dishonesty” and “grade point average”), (“academic dishonesty” and GPA), (“academic dishonesty” and grades)]. We also checked the articles published from January 1975 to January 2022 in the journals *Applied Cognitive Psychology, Educational Research Review, European Journal of Personality, Higher Education, Human Performance, International Education Studies, International Journal for Education Integrity, International Journal of Educational Psychology, Journal of Academic Ethics, Journal of Personality Assessment, Journal of Research in Personality, Learning and Individual Differences, Personality and Individual Differences, Research in Higher Education, Research in Higher Education Journal, Studies in Science Education*, and *The Journal of Higher Education*. Besides, the *International Journal of Educational Research* was examined from 2006, and the *College Student Journal* from 1996. We also examined the reference section of the following literature reviews on CAB: Wrightsman (1959), Bushway and Nash (1977), Ford and Richardson (1994), Crown and Spiller (1998), Whitley (1998), Cizek (1999), Credé et al. (2010), Giluk and Postlethwaite (2015), Paulhus and Dubois (2015), Gubbels et al. (2019), and Cuadrado et al. (2021). Last, we contacted a number of researchers on the topic with the purpose of including additional studies.

### Inclusion and Exclusion Criteria

In order to decide whether the primary studies could be included in the database, we used the following of criteria. First, they had to provide an effect size of the CAB-AP relationship or data for its calculation. Second, the CAB variable had to assess counterproductive behaviors performed by students in academic settings. Any measure assessing opinions, perceptions, intentions, or attitudes on CAB was not considered for this research. Moreover, the studies assessing absenteeism behaviors in samples composed of elementary school students were excluded due to the very probable justified reason of the absence. Likewise, in academic levels apart from elementary school, we excluded the studies in which the absences were justified (e.g., medical reasons) and/or reported by parents or guardians. Third, given that self-reported performance measures are highly consistent with those obtained from official records (Kuncel et al., 2005a), both types of AP measures were accepted. However, we excluded the cases where CAB was measured some time later than AP, for instance, when CAB was evaluated in higher education and AP in high school.

Following the PRISMA guidelines (Moher et al., 2009), Figure 1 presents a flow diagram with the number of studies considered in each phase of the search. By examining the studies provided by the sources described above, we obtained 11,760 references that were individually examined. Once duplicated studies were excluded, 6,710 studies were screened, of which 217 were fully assessed for eligibility. The last phase of the search yielded 206 studies that could be included in the meta-analysis with 231 independent samples and an accumulated sample size of 127,269 individuals.

**Figure 1:**
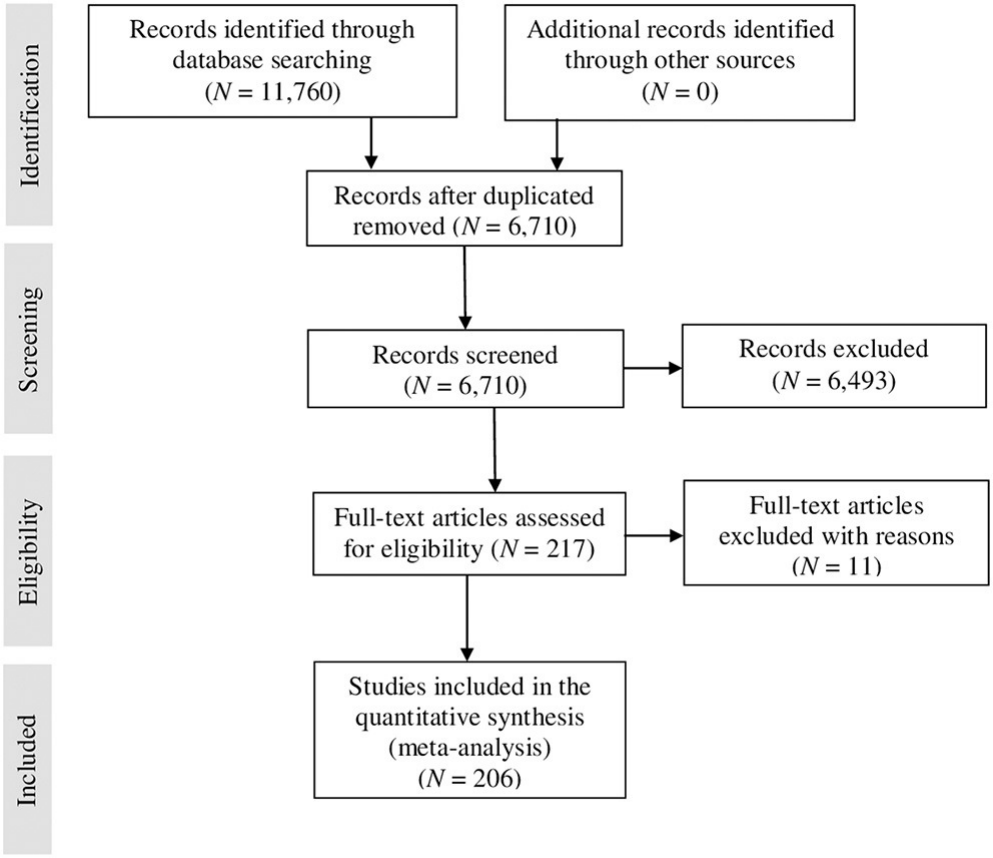
PRISMA flow diagram of the included and excluded studies.

### Coding Procedure

A database was created in order to register the studies' most relevant information. First, the characteristics of the study were coded [i.e., author(s), year of publication, title, type of publication]. Then, the characteristics of the sample were computed. We registered the country, the initial and final sample size, the academic level, and the academic discipline. Next, information regarding the CAB and AP variables was included. We coded the definition of the variables, the instrument used for their evaluation and its reliability, and the dichotomization of the variable. Last, information on the effect size or data for its calculation was recorded.

Next, we applied the following rules for the treatment of duplicated samples and of conceptual replications. If sample duplication was confirmed, we directly excluded the duplicated studies when, also, the involved variables were the same. However, we included all the relationships and integrated the smaller sample size when the variables differed among the studies. Additionally, when the variables were part of the same construct, the suggestions made by Schmidt and Hunter (2015) for the treatment of conceptual replications were followed. In these cases: (1) when the correlations among the variables of the conceptual replication were reported, we calculated the correlation for the composite following the procedure developed by Schmidt and Hunter (2015). We also calculated the reliability coefficient for the composite using the formula for composites provided by the same authors. When two or more indicators of AP were reported in the same study, they were treated as conceptual replications following the steps described above. However, if a general measure of AP (e.g., GPA) was provided together with more specific measures (e.g., course grade), only the first was included for being more perfect in terms of construct; (2) when the correlations between the variables of the conceptual replication were not provided, the average effect size was calculated. Even so, the different variables involved in the composite, whether averaged or excluded due to a more perfect variable in terms of construct existing, were individually coded for the purpose of carrying out the analyses of moderators. The same procedure was followed for the CAB measures.

The data of the primary studies included in the meta-analyses can be found on https://osf.io/27k3b/?view_only=7a16d4fd380e4b9eb7d6da877a0bbce6.

### Moderator Variables

In order to test potential moderator variables, the set of studies was broken down according to: (1) the type of AP measure [i.e., GPA, final course grade, examination(s) grade, and a miscellaneous category involving other AP measures]; (2) the type of source used to obtain the AP indicator (i.e., official sources vs. self-reported) and (3) the students' academic level (i.e., higher education, secondary education, and in elementary school).

We also calculated the extent to which the moderator variables overlapped. For this, we created a table reflecting the variables tested in each individual study. The next step was to calculate the Phi coefficient for each 2 × 2 combination of the categories between each pair of moderators. In total, 98 Phi coefficients were calculated. Later, the average Phi and the *SD* were computed for each pair of combined moderators. The results showed that moderators are virtually independent from each other (see Supplementary Table 6).

### Methods of Meta-Analysis and Effect Size Estimation

Psychometric methods of meta-analysis developed by Schmidt and Hunter (2015), which correct for the effects that artifactual errors exert on the mean effect size (underestimation of its magnitude and introduction of artifactual variability), were applied. For this, we used the meta-analysis software created by Schmidt and Le (2014). Corrections for measurement error in the predictor and criterion and indirect range restriction in the predictor variable we performed to calculate the true correlation (ρ). The operational correlation (*r*_o_) was also computed.

Effect sizes provided by the individual studies were directly considered for the analyses when they were expressed as a coefficient of the Pearson family (i.e., *r*, ϕ, Spearman's ρ). In other cases, a Cohen's *d* could be calculated, from which a Pearson correlation was then computed. The same strategy was followed when other statistics were reported (e.g., *F, t*, χ^2^, *z*). Correlational effect sizes were also corrected for dichotomization using the appropriate formulas (see Guilford and Fruchter, 1984). Lastly, the directionality of the relationships was adjusted as needed so that the scores on the variables represent higher levels of counterproductivity and performance.

### Outliers Identification and Risk of Bias

Similar to that which occurs in primary research, meta-analyses can be affected by the potential existence of extreme datapoints (Huffcutt and Arthur, 1995; Schmidt and Hunter, 2015; Webster, 2019). Considering that the effect sizes are randomly arranged around the mean effect size, our strategy for detecting potential outliers was to estimate the units of standard deviation that the coefficient reported by each primary study separated from the mean effect size of each meta-analysis (see Wilcox, 2014). We treated an individual study as an outlier when three or more units of standard deviation separated its effect size from the mean effect size. The analyses yielded eight potential outliers that were removed from the analyses in order to test their influence on the outcomes. The results indicated that the removal of the outliers had no impact on the mean effect sizes or their variability. For this reason, the meta-analyses were carried out including the outliers.

A cumulative meta-analysis (CMA) was also performed to assess the risk of bias. CMA is a meta-analysis run with one study, then repeated with a second study added. It is then repeated again with a third, and so on and so forth. This mechanism can be used to evaluate the risk of bias on a given meta-analysis (Schmidt and Hunter, 2015). The individual sample sizes are sorted according to their size, from the largest to the smallest, and a cumulative meta-analysis is carried out with the addition of each study. Next, we created a moving forest plot (see Supplementary Figure 1). The forest plot supports the absence of bias when a sustained line is observed after the addition of some validity coefficients. Supplementary Figure 1 shows the forest plot produced by CMA which represents the point estimate and the confidence interval for every new meta-analysis conducted with the addition of every single study. As can be seen, CMA shows that risk of bias is not a problem in the current meta-analysis because the line clearly stabilized with the progressive addition of new studies.

### Artifacts Distributions

#### Predictor Reliability

The reliability data reported in the primary studies was used to estimate the reliability of the predictor variable (CAB). The empirical distribution consisted of 67 internal consistency coefficients which had a mean value of α = 0.81 (*SD* = 0.09), ranging from 0.51 to 0.90. Giluk and Postlethwaite (2015) found a similar mean coefficient in their meta-analysis (α = 0.83, *K* = 11, *N* = 3,448). The reliability distributions for the CAB facets ranged from α = 0.80 (*SD* = 0.10) for low effort to α = 0.69 (*SD* = 0.12) for deception.

#### Criterion Reliability

Reliability for the criterion variable (AP) was also estimated using a distribution of the coefficients reported in the primary studies. Since estimates of AP reliability are rarely calculated and published, the empirical distribution was only composed of nine coefficients that ranged from 0.77 to 0.98. The mean value was α = 0.87 (*SD* = 0.07). This result is slightly lower than that found by Beatty et al. (2015) (α = 0.93, *N* = 818,179 subjects) and a little higher than that reported in the meta-analysis by Salgado and Táuriz (2014) (α = 0.80, *SD* = 0.10, *K* = 6). The reliability distributions for the different AP indicators ranged from α = 0.88 (*SD* = 0) for course grades to α = 0.72 (*SD* = 0.02) for the miscellaneous category.

#### Predictor Range Restriction

Indirect range restriction was corrected in the predictor variable (CAB). Range restriction coefficients were estimated using the formula derived by Schmidt et al. (1976) based on the selection ratio. For this, we used the software VALCOR developed by Salgado (1997). The average coefficient of the resulting distribution was *u* = 0.72 (*K* = 104, *SD* = 0.14). Thus far, no meta-analysis on CAB has ever applied correction for range restriction in this variable and, consequently, there is no previous data that allows comparisons to be made regarding the magnitude of this value. The distributions of range restriction coefficients for the CAB facets ranged from *u* = 0.88 (*SD* = 0) for course grades to α = 0.72 (*SD* = 0.02) for the miscellaneous category.

## Results

### Meta-Analysis of the Combination of CAB and Its Facets With AP

Table 2 shows the meta-analysis results for the combination of CAB and its facets with AP. From left to right, we present the total sample size (*N*), the number of independent samples (*K*), the sample size weighted observed correlation (*r*), the variance and the standard deviation of observed effect sizes (*S*${}_{r}^{2}$ and *SD*_*r*_), the sampling error variance (SEV), the operational and the true effect sizes (*r*_o_ and ρ), the standard deviation of ρ (*SD*_ρ_), the % of variance due to artifactual errors (%VE), and the upper and lower limits of the 80% credibility interval (80% CrI_ρ_) and of the 90% confidence interval of ρ (CI_ρ_ = 90%).

**Table 2:** Meta-analyses of the combination of overall CAB, its facets, and AP.

											**80% CrI**_**ρ**_		**90% CI**_**ρ**_	
	***N***	***K***	***r***	***S${}_{r}^{2}$***	***SD_***r***_***	**SEV**	***r*** **_o_**	**ρ**	***SD*** **_ρ_**	**%VE**	**LL**	**UL**	**LL**	**UL**
**Overall CAB-AP**	127,269	231	−0.25	0.0405	0.2012	0.0016	−0.38	−0.40	0.2701	14	−0.74	−0.05	−0.42	−0.38
Absenteeism	69,453	117	−0.29	0.0326	0.1806	0.0014	−0.45	−0.48	0.2330	24	−0.78	−0.19	−0.50	−0.46
Low effort	45,296	41	−0.19	0.0377	0.1940	0.0008	−0.28	−0.30	0.2754	8	−0.65	0.06	−0.35	−0.25
Cheating	11,000	26	−0.11	0.0094	0.0968	0.0023	−0.19	−0.21	0.1334	37	−0.38	−0.03	−0.24	−0.18
Deception	5,959	18	−0.20	0.0279	0.1671	0.0028	−0.28	−0.31	0.2206	23	−0.60	−0.03	−0.37	−0.25
Breach of rules	8,648	11	−0.15	0.0255	0.1599	0.0012	−0.21	−0.23	0.2261	6	−0.52	0.06	−0.31	−0.15
Plagiarism	5,979	15	−0.11	0.0117	0.1082	0.0025	−0.20	−0.21	0.1646	25	−0.42	0.00	−0.25	−0.17
Misuse of resources	1,040	3	−0.08	0.0094	0.0971	0.0029	−0.11	−0.12	0.1230	30	−0.28	0.04	−0.21	−0.03
**Results by the type of AP measure**														
GPA	75,176	137	−0.23	0.0281	0.1677	0.0017	−0.35	−0.37	0.2264	18	−0.65	−0.08	−0.39	−0.34
Course grade	44,136	59	−0.29	0.0614	0.2478	0.0011	−0.40	−0.42	0.3210	5	−0.83	−0.01	−0.47	−0.36
Examination (s)	10,157	48	−0.36	0.0162	0.1273	0.0036	−0.58	−0.63	0.0548	90	−0.70	−0.56	−0.65	−0.61
Miscellaneous	7,510	28	−0.26	0.0229	0.1515	0.0033	−0.43	−0.45	0.1952	32	−0.70	−0.20	−0.49	−0.41
**Results by the source of AP measure**														
Official source	65,646	126	−0.28	0.0534	0.2311	0.0017	−0.40	−0.42	0.3026	13	−0.81	−0.04	−0.45	−0.39
Self-reported	57,231	90	−0.23	0.0252	0.1586	0.0014	−0.35	−0.37	0.2157	18	−0.65	−0.09	−0.39	−0.34
**Results by the educational level**														
Higher education	62,823	193	−0.27	0.0448	0.2117	0.0027	−0.42	−0.44	0.2847	18	−0.80	−0.08	−0.46	−0.42
High school	61,615	33	−0.23	0.0365	0.1909	0.0005	−0.33	−0.35	0.2610	6	−0.69	−0.02	−0.40	−0.30
Elementary school	2,300	4	−0.40	0.0034	0.0580	0.0012	−0.50	−0.52	0.0535	38	−0.59	−0.45	−0.57	−0.47

The first row of the table shows the results for the broader category of analyses. We found a moderate true correlation of ρ = −0.40 for the CAB-AP relationship. The total sample size was 127,269 students and the number of independent samples was 231. Neither the confidence interval nor the credibility interval included zero. However, given the small percentage of variance explained by artifactual errors (%VE = 14%) and the amplitude of the credibility interval (90% CV = −0.05), it appears to be appropriate to conduct moderator analyses. The results for the CAB facets showed a negative relationship between CAB and AP regardless of the facet considered. The effect sizes ranged from ρ = −0.48 for absenteeism to ρ = −0.12 for misuse of resources. The percentage of explained variance by the artifactual errors ranged from 6% for breach of rules to 37% for cheating. The confidence intervals excluded zero for every CAB facet and there was generalization of the results for absenteeism (ρ = −0.48), deception (ρ = −0.31), and cheating (ρ = −0.21) due to the fact that the 90% credibility intervals did not include zero. These findings support hypothesis 1.

### Moderator Effects of the Type of AP Measure, the Source of the AP Measure, and the Educational Level on the Relationship Between CAB and AP

The first moderator tested was the type of measure used to assess AP. As can be seen in Table 2, the true correlations were negative for all the cases, ranging from ρ = −0.63 for examination grades to ρ = −0.37 for GPA. The confidence intervals and the credibility intervals excluded zero regardless of the AP measure used. The results also show that the true correlation increases in magnitude as the AP measures decrease in amplitude (the effect sizes were ρ = −0.37 for GPA, ρ = −0.42 for final course grades, and ρ = −0.63 for examination grades).

The second moderator was the source of the AP measure. The results show that the relationship between CAB and AP is moderate and negative despite of the source considered (ρ = −0.42 for measures obtained from official sources and ρ = −0.37 for self-reported measures). The confidence and the credibility intervals excluded zero.

The third moderator was the academic level of students. The true correlations ranged from ρ = −0.35 when CAB was related to AP using high school samples, to ρ = −0.52 for samples of students enrolled in elementary school. Both the confidence and the credibility intervals excluded zero regardless of the academic level considered.

### Hierarchical Meta-Analyses

In addition to the moderator analyses, we have also carried out hierarchical meta-analyses whenever possible. With this aim, the studies were disassembled by one key moderator variable, and then recombined and subsequently taken apart again by another key moderator variable (Schmidt and Hunter, 2015, p. 381). Thus, three sets of hierarchical meta-analyses are presented. First, Table 3 shows the meta-analysis of the relationship between CAB and AP according to the type of AP measure and to the AP measure's source. As can be seen, when AP is reflected as the students' GPA, the results are very similar both for the official and for the self-reported measures (ρ = −0.34 and ρ = −0.38, respectively). The credibility and the confidence intervals exclude zero.

**Table 3:** Hierarchical meta-analyses of the CAB-AP relationship according to the type of AP measure and its source.

											**80% CrI**_**ρ**_		**90% CI**_**ρ**_	
	***N***	***K***	***r***	***S${}_{r}^{2}$***	***SD_***r***_***	**SEV**	***r*** **_o_**	**ρ**	***SD*** **_ρ_**	**%VE**	**LL**	**UL**	**LL**	**UL**
**GPA**														
Official source	19,142	49	−0.22	0.0343	0.1852	0.0023	−0.33	−0.34	0.2438	16	−0.65	−0.03	−0.38	−0.30
Self-reported	53,501	79	−0.23	0.0252	0.1588	0.0013	−0.36	−0.38	0.2231	14	−0.67	−0.10	−0.41	−0.35
**Course grade**														
Official source	43,628	56	−0.29	0.0620	0.2489	0.0011	−0.41	−0.43	0.3243	5	−0.84	−0.01	−0.48	−0.37
**Examination (s)**														
Official source	9,764	46	−0.37	0.0157	0.1252	0.0036	−0.57	−0.62	0.0783	78	−0.72	−0.52	−0.64	−0.60
Self-reported	434	2	−0.22	0.0099	0.0996	0.0042	−0.24	−0.27	0.0910	42	−0.39	−0.15	−0.39	−0.15
**Miscellaneous**														
Official source	3,388	16	−0.30	0.0189	0.1373	0.0040	−0.43	−0.48	0.1752	25	−0.70	−0.25	−0.54	−0.42
Self-reported	3,100	9	−0.16	0.0135	0.1160	0.0028	−0.28	−0.29	0.1632	33	−0.50	−0.08	−0.35	−0.23

When AP is measured as examination grades, the results show a different magnitude according to the source of the AP indicator. When official records are used, the effect size is ρ = −0.62. When self-reports are analyzed, the true effect size is lower (ρ = −0.27). However, these findings must be taken with caution since the number of effect sizes included for the meta-analysis with the self-reported measures is very small.

In regard to the miscellaneous category of AP measures, the results appear to be more robust for the official than for the self-reported measures. The true effect sizes were ρ = −0.48 and ρ = −0.29, respectively. In both cases, the credibility and the confidence intervals excluded zero. Again, the analyses regarding the self-reported measures were conducted with a small number of primary studies. Last, it was not possible to carry out a comparison between the meta-analyses concerning the course grades due to the fact that only AP measures obtained from official sources were available.

Table 4 displays the results of the meta-analysis for the relationship between CAB and AP according to the CAB facets and to the type of AP measure. As noted, the relationship between some specific CAB facets and the different types of AP measures could not be examined due to the lack of primary studies. The only two facets that could be associated with every measure of AP were absenteeism and low effort. In the case of absenteeism, the true effect sizes were ranged from ρ = −0.39 for GPA, −0.56 for course grades, and ρ = −0.69 for examination grades. In the three cases, the confidence and the credibility intervals excluded zero. The results showed that the magnitude of the effect sizes increased as the AP measure decreased in amplitude (ρ = −0.39 for GPA, ρ = −0.56 for course grades, and ρ = −0.69 for examination grades). Additionally, absenteeism was the CAB facet most strongly related to course grades, examination grades, and the miscellaneous category of AP.

**Table 4:** Hierarchical meta-analyses of the CAB-AP relationship according to the CAB facets and to the type of AP measure.

											**80% CrI**_**ρ**_		**90% CI**_**ρ**_	
	***N***	***K***	***r***	***S${}_{r}^{2}$***	***SD***_***r***_	**SEV**	***r*** **_o_**	**ρ**	***SD*** **_ρ_**	**%VE**	**LL**	**UL**	**LL**	**UL**
**GPA**
Absenteeism	37,924	55	−0.22	0.0176	0.1325	0.0013	−0.37	−0.39	0.1784	33	−0.62	−0.16	−0.41	−0.36
Low effort	21,144	30	−0.32	0.0298	0.1726	0.0012	−0.44	−0.47	0.1831	35	−0.70	−0.23	−0.51	−0.42
Cheating	9,305	21	−0.10	0.0078	0.0885	0.0022	−0.17	−0.18	0.1237	40	−0.34	−0.02	−0.21	−0.15
Deception	2,336	7	−0.16	0.0052	0.0718	0.0029	−0.27	−0.30	0	100	−0.30	−0.30	−0.34	−0.27
Breach of rules	6,670	8	−0.10	0.0181	0.1345	0.0012	−0.16	−0.17	0.2058	7	−0.43	0.09	−0.25	−0.09
Plagiarism	5,205	11	−0.12	0.0096	0.0979	0.0021	−0.20	−0.22	0.1360	31	−0.39	−0.04	−0.26	−0.17
Misuse of resources	1,040	3	−0.08	0.0094	0.0971	0.0029	−0.11	−0.12	0.1230	30	−0.28	0.04	−0.21	−0.03
**Course grade**														
Absenteeism	28,392	44	−0.37	0.0383	0.1958	0.0012	−0.53	−0.56	0.2359	10	−0.86	−0.26	−0.61	−0.51
Low effort	22,734	8	−0.07	0.0159	0.1261	0.0004	−0.11	−0.11	0.1919	3	−0.36	0.13	−0.18	−0.04
Deception	2,378	5	−0.14	0.0374	0.1935	0.0020	−0.16	−0.16	0.2191	5	−0.44	0.12	−0.30	−0.02
Breach of rules	1,805	3	−0.34	0.0175	0.1322	0.0013	−0.40	−0.42	0.1500	9	−0.61	−0.23	−0.55	−0.29
Plagiarism	429	2	0.04	0.0222	0.1488	0.0047	0.05	0.06	0.2007	21	−0.20	0.31	−0.23	0.12
**Examination (s)**														
Absenteeism	9,373	39	−0.36	0.0147	0.1211	0.0032	−0.62	−0.69	0	100	−0.69	−0.69	−0.71	−0.67
Low effort	750	6	−0.06	0.0080	0.0894	0.0080	−0.12	−0.12	0	100	−0.12	−0.12	−0.18	−0.06
Deception	639	7	−0.43	0.0206	0.1437	0.0074	−0.46	−0.53	0.1381	36	−0.70	−0.35	−0.62	−0.43
**Miscellaneous**														
Absenteeism	3,093	16	−0.35	0.0190	0.1362	0.0040	−0.55	−0.61	0.1265	56	−0.77	−0.44	−0.65	−0.56
Low effort	1,571	5	−0.23	0.0066	0.0814	0.0029	−0.35	−0.37	0.0909	47	−0.49	−0.26	−0.43	−0.31
Cheating	1,178	3	−0.15	0.0001	0.0122	0.0024	−0.28	−0.28	0	100	−0.28	−0.28	−0.33	−0.24
Plagiarism	707	3	−0.03	0.0162	0.1274	0.0043	−0.03	−0.03	0.1216	26	−0.18	0.13	−0.15	0.09

In the case of low effort, the largest true effect size was ρ = −0.47 for GPA, being the CAB facet most strongly associated with this indicator of AP. The lowest was ρ = −0.11 for course grades. This was the only case in which generalization of the results did not occur. Confidence intervals excluded zero in every case. The remaining CAB facets were only examined for some types of AP measures. Cheating, deception, breach of rules, plagiarism, and misuse of resources yielded true effect sizes with GPA ranging from ρ = −0.12 for misuse of resources to ρ = −0.30 for deception. Generalization of the results occurred in all the cases except for breach of rules. Concerning course grades, only deception, breach of rules, and plagiarism were analyzed. The results ranged from ρ = −0.42 for breach of rules to ρ = 0.06 for plagiarism. There was generalization of the results for breach of rules and the confidence intervals excluded zero for all the tested relationships except for that concerning plagiarism. Nevertheless, these results should be carefully interpreted due to the small number of studies in some cases.

Besides absenteeism and low effort, deception was the only CAB facet whose association was tested with examination grades. The result was ρ = −0.53 and the confidence and the credibility intervals excluded zero. Finally, the relationships of the miscellaneous category of AP with cheating and plagiarism yielded an effect size of ρ = −0.28 and ρ = −0.03, respectively. These results should also be taken with caution due to the very small number of primary studies accumulated.

Table 5 presents the results of the CAB-AP relationship according to the CAB facets and to the students' academic level. The results show that the type of counterproductive behavior with the strongest correlation in higher education is absenteeism, with a true effect size of ρ = −0.62. With respect to the other CAB facets, the results ranged from ρ = −0.26 for deception to ρ = −0.11 for misuse of resources. Confidence intervals excluded zero in all the cases and generalization of the results occurred for absenteeism, low effort, cheating, and breach of rules.

**Table 5:** Hierarchical meta-analyses of the CAB-AP relationship according to the CAB facets and to the academic level.

											**80% CrI**_**ρ**_		**90% CI**_**ρ**_	
	***N***	***K***	***r***	***S${}_{r}^{2}$***	***SD_***r***_***	**SEV**	***r*** **_o_**	**ρ**	***SD*** **_ρ_**	**%VE**	**LL**	**UL**	**LL**	**UL**
**Higher education**														
Absenteeism	32,143	105	−0.39	0.0467	0.2162	0.0024	−0.57	−0.62	0.2356	28	−0.92	−0.31	−0.65	−0.58
Low effort	9,306	33	−0.13	0.0091	0.0955	0.0035	−0.21	−0.23	0.1056	55	−0.36	−0.09	−0.25	−0.20
Cheating	9,986	24	−0.10	0.0074	0.0861	0.0024	−0.18	−0.19	0.1172	43	−0.34	−0.04	−0.22	−0.16
Deception	4,209	11	−0.15	0.0301	0.1736	0.0025	−0.23	−0.26	0.2610	20	−0.59	0.07	−0.34	−0.18
Breach of rules	1,451	5	−0.13	0.0055	0.0744	0.0033	−0.19	−0.22	0.0744	60	−0.31	−0.12	−0.27	−0.16
Plagiarism	5,911	14	−0.11	0.0114	0.1068	0.0023	−0.18	−0.19	0.1519	27	−0.39	0.00	−0.24	−0.15
Misuse of resources	1,040	3	−0.08	0.0094	0.0971	0.0029	−0.10	−0.11	0.1150	30	−0.26	0.03	−0.21	−0.02
**High school**														
Absenteeism	37,307	12	−0.20	0.0046	0.0678	0.0003	−0.33	−0.35	0.0766	49	−0.45	−0.25	−0.37	−0.33
Low effort	35,877	7	−0.21	0.0435	0.2085	0.0002	−0.28	−0.29	0.2802	3	−0.65	0.07	−0.42	−0.16
Cheating	1,014	2	−0.25	0.0098	0.0989	0.0017	−0.27	−0.29	0.1018	18	−0.42	−0.16	−0.40	−0.17
Deception	818	5	−0.27	0.0017	0.0409	0.0053	−0.27	−0.31	0	100	−0.31	−0.31	−0.37	−0.26
Breach of rules	5,942	5	−0.10	0.0191	0.1382	0.0008	−0.14	−0.15	0.1978	5	−0.40	0.10	−0.25	−0.05
**Elementary school**														
Deception	932	2	−0.35	0.0002	0.0155	0.0016	−0.41	−0.41	0	100	−0.41	−0.41	−0.47	−0.36

In high school, the CAB facets reaching the largest true effect size were, as it occurred for higher education, absenteeism and deception. The results were ρ = −0.35 and ρ = −0.31, respectively. For the remaining facets, the effect sizes ranged from ρ = −0.29 for low effort and cheating and to ρ = −0.15 for breach of rules. In all the cases, confidence intervals excluded zero. The credibility intervals included zero for low effort and breach of rules.

Last, the only CAB facet whose relationship was examined with AP in elementary school was deception. The result was ρ = −0.41, and both the credibility and the confidence intervals excluded zero.

### Meta-Analytic Path Analysis Model

A path analysis was applied to test a model of the CAB-AP relationship, and the predictive weight of CAB, students' personality (i.e., the Big Five model dimensions of personality), and cognitive intelligence on AP. For this, the first step was to create a pooled matrix of meta-analytical correlations between the variables. Following the recommendations of Hoyle and Kenny (1999), Schmidt and Hunter (2015), and Fritz et al. (2016), the validity coefficients were corrected by measurement error in X and Y because measurement error violates the assumption of independence of errors and, consequently, can bias the estimation of parameters. The correlation matrix used for the analyses (see Table 6) includes the true correlation between overall CAB and AP reported in the current study. The meta-analyses providing the remaining coefficients composing the matrix were selected because: (1) they were performed using psychometric methods of meta-analysis with artifactual corrections or suitable to be corrected by artifacts, and (2) they are considered some of the highest quality studies that have been widely used to develop meta-analytic path analyses on the relationships among AP, the Big Five dimensions of personality, and intelligence.

**Table 6:** Matrix of meta-analytical correlations used for the path analysis (*N* = 12,087).

	**1**	**2**	**3**	**4**	**5**	**6**	**7**	**8**
1. CAB	–							
2. AP	−0.40^a^							
3. Emotional stability	−0.01^b^	0.02^c^						
4. Extraversion	0.02^b^	−0.01^c^	0.24^e^					
5. Openness to experience	−0.08^b^	0.13^c^	0.19^e^	0.45^e^				
6. Agreeableness	−0.14^b^	0.08^c^	0.42^e^	0.26^e^	0.17^e^			
7. Conscientiousness	−0.28^b^	0.25^c^	0.52^e^	0.17^e^	0.09^e^	0.39^e^		
8. Intelligence	−0.19^b^	0.68^d^	0.09^f^	0.02^f^	0.22^f^	0.00^f^	−0.04^f^	–

*CAB, counterproductive academic behaviors; AP, academic performance; ^a^data obtained in the current study (Table 2); ^b^meta-analytic coefficients reported in Cuadrado et al. (2021); ^c^meta-analytic coefficients reported in Poropat (2009) corrected for the current study by range restriction using u coefficients published by Salgado and Táuriz (2014); ^d^estimated coefficient from the effect sizes reported Postlethwaite (2011) for the relationships between AP and fluid intelligence measures (ρ = 0.43, K = 67, N = 7,991), crystallized intelligence measures (ρ = 0.71, K = 157, N = 199,642) and general intelligence measures (ρ = 0.74, K = 110, N = 29,739); ^e^meta-analytic data published by Mount et al. (2005); ^f^meta-analytic data published by Judge et al. (2007)*.

The results reported in the meta-analysis of Poropat (2009) were used to estimate the relationship between the Big Five dimensions and AP. In order to be included in the input matrix, we proceeded to correct these results for range restriction in the predictor variables using the coefficients published by Salgado and Táuriz (2014). Likewise, we used the effect sizes reported by Postlethwaite (2011) for the relationship between intelligence and AP. In this case, we calculated the average weighted effect size for the relationships of AP with fluid intelligence, crystallized intelligence, and general intelligence. We also included the results by Mount et al. (2005) for the relationships among the Big Five dimensions, those by Judge et al. (2007) for the relationships between intelligence and the Big Five dimensions, and those by Cuadrado et al. (2021) for the relationships of CAB with intelligence and with the Big Five dimensions of personality. Following the suggestions by Schmidt and Hunter (2015), the harmonic mean of the samples used in the estimation of the coefficients included in the input matrix was used as the model sample size to estimate the goodness-of-fit indexes. The result was *N* = 12,087 subjects.

The second step was to fit the path model to the matrix of meta-analytic correlations. For this purpose, we used the software LISREL (8.2) by Jöreskog and Sörbom (1998). The tested model included two criterion variables: (1) overall CAB, directly predicted by conscientiousness, agreeableness, and cognitive intelligence; and (2) AP, directly predicted by CAB, openness to experience, conscientiousness, and cognitive intelligence. The model also considered conscientiousness and cognitive intelligence as indirect predictors of AP through overall CAB.

The model showed a good fit to the data. The adjusted goodness of fit index (AGFI) and the non-normed fit index (NNFI) had a value of 0.99. The comparative fit index (CFI) and the normed fit index (NFI) had a value of 1.00. The indexes of absolute fit SRMR and RMSEA were 0.006 and 0.032, respectively. All of these indexes were on the acceptable ranges suggested by Hu and Bentler (1999) and Kline (2011). However, the most important fit index is the standardized root mean square residual (SRMR) because it indicates the degree of absolute adjustment of the model which is not affected by the sample size. Hu and Bentler (1999; see also Kline, 2011) found out that a value of SRMR ≤ 0.08 indicates an acceptable fit. Therefore, the combination of absolute fit index (i.e., SRMR, RMSEA) with the approximate fit indexes (i.e., AGFI, NNFI, CFI, and NFI) indicates a good fit for the model.

**Figure 2:**
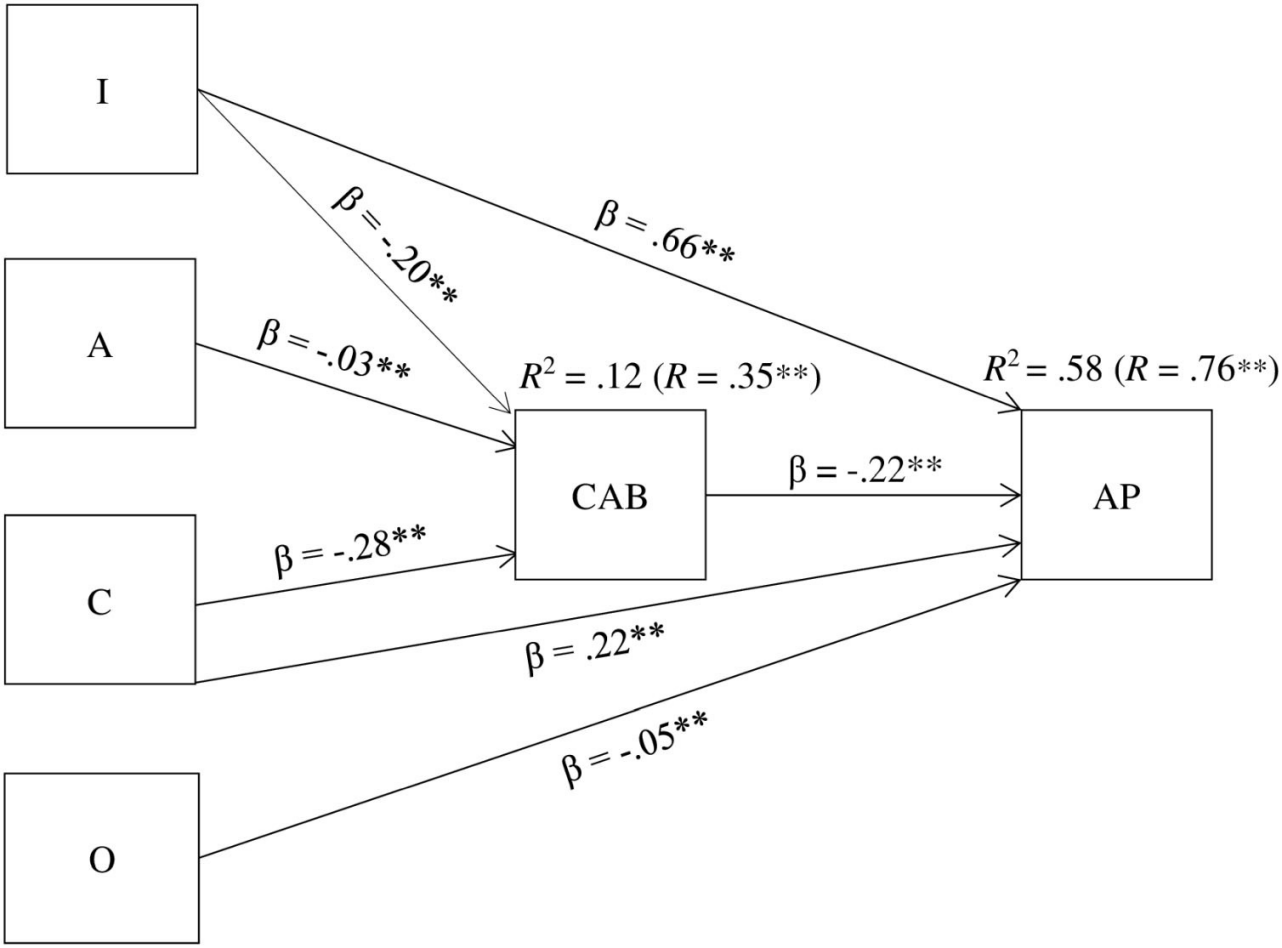
Graphical representation of the meta-analytic path analysis model.

The graphical representation of the model appears in Figure 2. As shown, agreeableness, conscientiousness, and cognitive intelligence had a significant negative relationship with overall CAB. Altogether, they accounted for 12% of CAB variance (*R* = 0.35, *p* < 0.001), being conscientiousness the best predictor with a beta value of β = −0.28 (*p* < 0.01), followed by intelligence (β = −0.20, *p* < 0.01). Among the variables that directly determined AP, cognitive intelligence appeared as the best predictor with a standardized weight of β = 0.66 (*p* < 0.01). The second-best predictor was conscientiousness with a beta value of β = 0.22 (*p* < 0.01). In the case of openness to experience, the effect size was small but significant (β = −0.05, *p* < 0.01). Last, CAB showed a negative relationship on AP with a beta of β = −0.22 (*p* < 0.01), partially explained by the effect of conscientiousness and cognitive intelligence. Altogether, the predictor variables accounted for the 58% of AP variance (*R* = 0.76, *p* < 0.001).

## Discussion

Decades of research have shown that CAB is a pervasive phenomenon in which a significant number of students have engaged with at some point in their studies. The severity of its consequences makes the empirical study of CAB a priority. The main goal of the present research was to further the knowledge on CAB by examining its relationship with students' AP. Specifically, this study has contributed to the scientific literature in several ways. The first contribution has been to present a theory of the CAB-AP relationship, which aims to overcome the atheoretical limitation of the previous research. This theory describes the structure of CAB as a three-stratum hierarchical structure and posits a number of propositions on the CAB antecedents as well as on the CAB effects on students' performance, teachers' motivation, institutions' climate and culture, and institutions' sustainability. This theory permitted that the empirical research conducted in this study was a theory-guided one. The theory also suggests some practical strategies to reduce the frequency and intensity of CAB and, by doing so, to improve students' AP.

The second contribution was to provide a robust and updated estimate of the true correlation between overall CAB and AP. Despite the publication of a previous meta-analysis on this relationship (i.e., Whitley, 1998), the current meta-analysis overcomes the limitations of the former study. The findings revealed a moderate and negative true correlation between overall CAB and AP, meaning that students who engage in academic counterproductivity tend to also demonstrate a poorer academic performance. Our estimates are consistent with the directionality of the results obtained by Whitley (1998), but the true effect reported in the present study (0.40) is larger in size. Some possible explanations for this might be: (1) a more accurate estimate of the relationship due to the higher number of studies meta-analyzed which, consequently, increased the total sample size and lowered the sampling error; and (2) a more accurate estimate of the true effect size and the true error due to the application of meta-analytical methods that corrected the observed coefficients for measurement error in X and Y and range restriction in X.

The third contribution has been to examine the relationships between each CAB facet and AP. Previous research on CAB lacked a fine-grained empirical analysis of its dimensions. These analyses could potentially yield different relationships with the criterion variables, and, consequently, lead to different conclusions and implications. Focusing exclusively on overall CAB can obscure the results according to the form of CAB considered. As stated above, absenteeism is the only facet whose relationship with AP has been previously meta-analyzed (see Credé et al., 2010; Gubbels et al., 2019). In addition to the improvement of both methodological and non-methodological aspects of the previous reviews, the meta-analyses on the relationships between the remaining CAB dimensions and AP contributed to the scientific knowledge on the CAB dimensionality and its nomological network. The results showed that all the facets are negatively correlated to AP and that the results are generalizable. The dimensions most strongly related to AP are absenteeism (−0.48), deception (−0.31), and low effort (−0.30).

The fourth contribution has been to clarify the moderating role of: (a) the type of AP measure; (b) the source of the AP measure; and (c) the educational level of students. First, the meta-analytic findings showed the importance of considering the different measures of AP in the examination of the CAB-AP relationship. Although the results are generalizable regardless of which type of AP measure is used, the magnitude of the effects differed among the indicators. The strongest estimate was a true correlation of −0.63 when AP was conceptualized as examination grades. Moderator analyses also showed that the relationship between CAB and AP is negative and moderate despite the source used to obtain the AP measure (official measures vs. self-reported measures). Last, the meta-analyses on the CAB-AP relationship according to the students' educational stage indicated that both constructs are negatively associated regardless of whether higher education, high school, or elementary school samples are considered. The relationship was especially strong in elementary school (−0.52) and in higher education (−0.44).

The fifth contribution has been to show that absenteeism appeared to be the type of counterproductive behavior most strongly related to AP expressed as course grades (−0.56), examination grades (−0.69), and other AP measures (−0.61). Absenteeism was also the CAB facet most strongly related to AP both in higher education (−0.62) and high school (−0.35). Low effort appeared to be the best predictor of GPA (−0.47).

A sixth contribution has been to test a path analysis model based on the propositions of the theory of CAB, on the results of this research, and on the findings of previous meta-analyses. The results showed that: (1) conscientiousness and cognitive intelligence are the strongest predictors of CAB, and that (2) conscientiousness, cognitive intelligence, and CAB, are crucial in the explanation of AP. Altogether, they account for 58% of the AP true variance. Given the importance of AP in research and applied contexts, the magnitude of this result has important implications. These findings also reinforce the advantages of considering individual characteristics such as conscientiousness and cognitive intelligence as predictors of relevant criteria in educational settings. The model suggests that the potential effects of cognitive intelligence and personality on AP are both direct and indirect (through CAB and its facets).

### Implications for Research and Practice

Our findings have implications for research and practice. From a theoretical perspective, the empirical results allowed us to test some of the key points of the theory previously presented. Specifically, the theoretical approach sustained that CAB produces lower academic outcomes on the basis of an ample number of reasons. The results of the meta-analysis supported this point. Not only overall CAB was negatively related to AP, but every facet predicted academic results regardless of the AP indicator, its source, or the academic level considered.

A second salient point of the theoretical approach was to posit that the facets and the general CAB factor are related to academic criteria such as academic performance. It also argued that, when the facets are related to the same criterion, they are not related to the same extent. The results of the meta-analyses supported this premise and highlighted the relevance of considering the multidimensionality of CAB as facets are related in different ways to AP. For example, absenteeism, deception, and low effort shared more variance with AP than plagiarism or misuse of resources.

The theoretical approach also stated that CAB is a complex phenomenon determined by a number of individual and contextual characteristics. Specifically, the theory stresses the importance of the students' personality traits and cognitive intelligence as antecedents of academic counterproductivity. The results of the path analysis model empirically backed this point and partly revealed CAB's nomological network. Researchers have extensively tried to determine the variables that explain students' academic failure and success. However, the true validity coefficients of these relationships had never been used with this purpose. For this reason, the results reported in the model have contributed to the advance of theory construction in the study of academic performance. Furthermore, future research should take into account that personality and cognitive intelligence indirectly affect AP through the effects of those variables on CAB and its facets.

Another implication for research is related to the source of the AP indicator (i.e., official source vs. self-reported). Our findings agree with the results of Kuncel et al. (2005a) in regard to the use of self-reported measures of AP as a reliable indicator of students' academic performance. Researchers can rely on information provided by students to carry out their studies on the CAB-AP relationship.

From an applied perspective, our results might be useful for practitioners in both academic and occupational settings. Regarding the first domain, empirical evidence has highlighted the importance of AP as a criterion for academic decision-making. As posited before, early AP has been shown to predict future academic success (Kuncel et al., 2001, 2005b; Robbins et al., 2004; Grossbach and Kuncel, 2011; Richardson et al., 2012; Westrick et al., 2021). For this reason, it becomes crucial to control any factor that might have a negative impact on students' academic outcomes. Our meta-analyses suggest that CAB and AP are negatively related. This finding must be considered by academic administrators as a basis to implement actions to reduce the occurrence of CAB. In particular, the results obtained in the moderator and the hierarchical meta-analyses are key to make more efficient decisions. For instance, overall CAB appeared to be more closely related to some AP measures (i.e., examination grades) than to others. This can help faculty reinforce certain classroom policies when, for instance, a decisive examination for students (e.g., a final examination) is approaching. The results also showed that absenteeism is strongly linked to AP regardless of the AP measure considered. Faculty and academic administrators can use these results to focus the available resources on reinforcing existing attendance policies or to implement new ones. Furthermore, the hierarchical meta-analyses' results suggest that, although CAB and AP are negatively related in all of the academic stages, some CAB facets, such as absenteeism, are especially associated to a lower performance in higher education. This suggests that higher academic institutions should make a greater effort to reduce absenteeism rates among their students. However, the analyses concerning the high school level showed that the differences between the effect sizes of the different CAB facets are smaller in magnitude. Hence, an applied policy against CAB as a broad construct would be appropriate in this case.

Based on the theoretical approach stated above and on the empirical findings, we suggest a series of potential interventions which can be useful. According to the theory, a double strategy based on the increment of the severity of punishment and the increment of CAB control would be an effective way to reduce CAB. Some studies found that fear of penalty together with a higher perception of the probability to get caught can be the best strategy to reduce CAB (e.g., Diekhoff et al., 1999; Vandehey et al., 2007). Additionally, a policy of lower CAB tolerance by instructors would be a third useful strategy. As we mentioned above, the increase of the punishment severity depends on the policies of institutions and teachers. Therefore, both institutions and instructors should align their actions in this respect. In parallel they should also increase the visibility of the discovered cases to discourage other students who would otherwise commit CAB.

With regard to the increment of CAB control, this may be done using a variety of strategies: (a) using different versions of an examination instead a unique test; (b) when possible, using oral examinations; (c) having pop quizzes; (d) increasing the control of absenteeism and reinforcing incentives that motivate students to attend class (e.g., adding a percentage to the final score); (e) using technology to conduct audits of exams, essays, and to protect the fairness of the evaluations. With regard to the reduction of teachers' level of permissiveness, academic institutions should motivate them to be less tolerant with CAB and to reinforce their authority. The reduction of instructors' tolerance might be also indirectly obtained by reinforcing peers' (e.g., other students) sense of justice and the importance of being honest in academic settings. Furthermore, both administrators and faculty should promote a climate of integrity. Empirical findings so far suggest that, although instructors and educational managers tend to frown on academic counterproductivity, efforts to confront this problem appear to be rather limited (McCabe, 1993; Christensen-Hughes and McCabe, 2006). Several studies found a series of reasons provided by teachers to justify their absence of action: for instance, difficulty to prove the instances of misconduct, lack of knowledge about the procedures for handling the incidents, the perceived ineffectiveness of the procedures, lack of time to take the necessary actions, reluctance to confront students, or the high levels of anxiety and stress involved (see Lipson and McGavern, 1993; McCabe, 1993; Whitley and Keith-Spiegel, 2002; Coren, 2011).

Our findings also have implications for the occupational domain—especially for recruitment practitioners. As posited earlier, meta-analytical evidence suggests that AP is a valid predictor of future occupational achievements and other criteria of success in life (Cohen, 1984; Hunter and Hunter, 1984; Dye and Reck, 1989; Roth et al., 1996; Roth and Clarke, 1998; Strenze, 2007; Salgado and Moscoso, 2019). Certainly, there is no large difference between the individual differences required to succeed at work and those needed to achieve satisfactory results in the classroom (Kuncel et al., 2004). For this reason and given that grades are a reliable measure of academic success (Kuncel et al., 2005a; Bacon and Bean, 2006), we can trust them as an empirically backed tool that should be used by recruitment professionals when they decide to hire someone. As posited earlier, AP measures are actually used in personnel selection. However, Roth and Bobko (2000) noted that there is not much information on the frequency of their use. Some surveys have suggested that grades (i.e., GPA) are indeed regularly considered in high stakes decision processes. For instance, the non-profit organization NACE (National Association of Colleges Employers, 2019) which forecasts the hiring intentions of American employers as they relate to new college graduates, found that 63% of the respondents (*N* = 150 organizations) would screen candidates from the class of 2020 by GPA. This report, therefore, illustrates that grades are commonly considered as a hiring criterion, at least, when it comes to hiring recent graduates. For this reason and considering that CAB is known to affect students' grades, it becomes even more crucial to prevent these negative behaviors from happening. Additionally, CAB has been shown to predict subsequent counterproductive work behaviors. That is to say, students engaging in deviant behaviors at school (i.e., cheating, plagiarism, absenteeism) are more prone to behave in such manner at their future jobs (i.e., misuse of time and resources, poor quality work, poor attendance). Although the research on this relationship is scarce, the existing results show a moderate to high association between the two constructs [see, for example, Sims (1993) or Nonis and Swift (2001)]. These findings are not surprising since CAB and CWB have been shown to have some common predictors. Among them, conscientiousness and agreeableness have shown to predict negative behaviors both in the classroom and in the workplace (see Berry et al., 2007; Giluk and Postlethwaite, 2015; Cuadrado et al., 2021). Hence, we would like to bring to the attention of human resources professionals the benefits of using personality measures in their hiring decisions as they have not only been shown to predict subsequent job performance, but also counterproductive work behaviors. In particular, we recommend using forced-choice personality measures with a quasi-ipsative format. This type of test has shown appropriate psychometric properties and also has been shown to be faking resistant (see Jackson et al., 2000; Christiansen et al., 2005; Otero et al., 2020; Martínez and Salgado, 2021; Martínez et al., 2021).

### Limitations of the Study and Suggestions for Future Research

This study has some limitations that need to be addressed in future research. First, the number of studies integrated was small for some hierarchical meta-analyses. In other cases, we were not able to carry out the meta-analyses due to the lack of primary studies. Consequently, we recommend carrying out more primary research in order to examine the specific associations that could not be examined or were examined with few studies.

We also acknowledge the relatively large true variance reported in some of the results. Although the moderator analyses and the hierarchical meta-analyses contributed to the clarification of the true variance's magnitude in some cases, it was still large for some relationships. Thus, it becomes necessary to examine additional moderator variables. Among these variables, some contextual influences that have been shown to predict CAB could moderate the CAB-AP relationship and help reduce the true variance. Some examples could be the existence of honor codes, peers' disapproval of CAB, peers' engagement in CAB, the classroom environment, or students' membership in fraternities or sororities (see, for instance, McCabe and Treviño, 1993, 1997; Pulvers and Diekhoff, 1999; McCabe et al., 2002, 2006; Storch and Storch, 2002). The country where the individual studies were performed should be also tested as a potential moderating variable. Although CAB is a universal phenomenon happening in every region, no systematic research has yet examined whether the country or cultural values could moderate the CAB-AP relationship.

Last, this study has permitted us to partially test the theoretical approach presented above. However, some of the key points posited by the theory still need to be supported by empirical evidence. We strongly encourage researchers to make a contribution on the theory construction of CAB by testing the role of the prospect theory in CAB's occurrence, or the effectiveness of different control mechanisms of this phenomenon.

## Data Availability Statement

The original contributions presented in the study are included in the article/Supplementary Material, further inquiries can be directed to the corresponding author/s.

## Author Contributions

All authors have made a substantial, direct, and equal contribution to this research.

## Funding

This research was partially supported by grants PSI2017-87603-P, PID2020-114984GB-I00, and PID116409GB-I00 from the Spanish Ministry of Science and Innovation.

## Conflict of Interest

The authors declare that the research was conducted in the absence of any commercial or financial relationships that could be construed as a potential conflict of interest.

## Publisher's Note

All claims expressed in this article are solely those of the authors and do not necessarily represent those of their affiliated organizations, or those of the publisher, the editors and the reviewers. Any product that may be evaluated in this article, or claim that may be made by its manufacturer, is not guaranteed or endorsed by the publisher.
