# Supplementary material for: Counterproductive Academic Behaviors and Academic Performance: A Meta-Analysis and a Path Analysis Model
Source: Front Psychol. 2022 Jun 2;13:893775. doi: 10.3389/fpsyg.2022.893775 (PMC9200985; doi:10.3389/fpsyg.2022.893775)

frontiers

Supplementary Material

Table S1. Meta-analytic Matrix of the Observed Intercorrelations Among the CAB Facets

	1	2	3	4	5	6
1. Absenteeism	-					
2. Low effort	.52					
3. Cheating	.39	.28				
4. Deception	.41	.33	.34			
5. Breach of rules	.29	.35	.28	.39		
6. Plagiarism	.32	.31	.25	.34	.41	
7. Misuse of resources	.38	.31	.27	.36	.27	.35

Note. Meta-analyses of the primary data reported by Cuadrado (2018), Martínez (2019), and Salgado (2014); K = 4 and N = 2,074 for all the cases.

Table S2. Factorial Loadings of the Confirmatory Factorial Analysis Calculated Using the Matrix Presented in Table S1

CAB facet	Factor 1
Absenteeism	.678
Low effort	.610
Cheating	.509
Deception	.624
Breach of rules	.584
Plagiarism	.534
Misuse of resources	.550

Table S3. Fit Indexes for One Factor Solution (CFA)

χ^2	df	AGFI	NNFI	CFI	SRMR	RMSEA
281.06	14	.94	.89	.93	.040	.088

Note. Acceptable values for the fit indexes are CFI > .90, SRMR < .08, RMSEA < .10 (Hu & Bentler, 1999; Kline, 2011).

Table S4. Factorial Loadings of the Exploratory Factorial Analysis Calculated Using the Matrix Presented in Table S1 and Fit Indexes for One Factor Solution

CAB facet	Factor 1
Absenteeism	.685
Low effort	.621
Cheating	.512
Deception	.618
Breach of rules	.552
Plagiarism	.549
Misuse of resources	.549

Table S5. Fit Indexes for One Factor Solution (EFA)

χ^2	df	AGFI	NNFI	CFI	SRMR	RMSEA
243.25	14	.99	.93	.96	.047	.089

Note. Acceptable values for the fit indexes are CFI > .90, SRMR < .08, RMSEA < .10 (Hu & Bentler, 1999; Kline, 2011).

Table S6. Average Phi Correlations for the Combinations between Moderators

	CAB facet - AP measure	CAB facet - educational level	AP measure - educational level	CAB facet - AP source	Educational level - AP source	AP measure - AP source
Mean Φ	-.01	.00	-.00	.00	.00	.00
SD	.15	.11	.09	.27	.13	.36

Figure S1. Forest Plot CMA for the CAB-AP Relationship

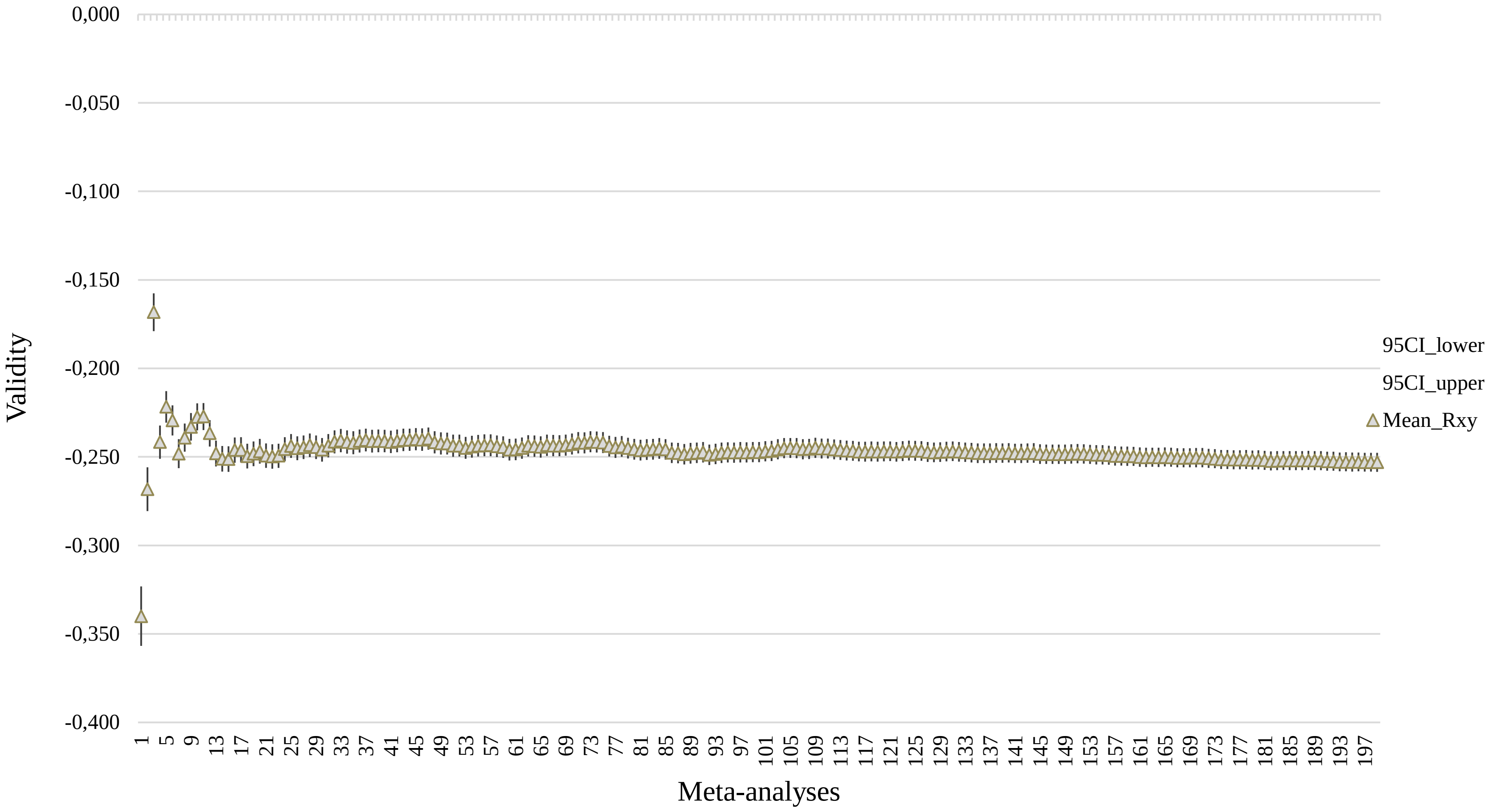

Supplement: Supplementary file 1 [file Data_Sheet_1.PDF]
